# Supplementary material for: Temporal Microbial Dynamics in Feces Discriminate by Nutrition, Fecal Color, Consistency and Sample Type in Suckling and Newly Weaned Piglets
Source: Animals (Basel). 2023 Jul 9;13(14):2251. doi: 10.3390/ani13142251 (PMC10376145; doi:10.3390/ani13142251)
Supplement: Supplementary file 1 [file animals-13-02251-s001.zip › animals-2464738-new supplementary.pdf]

animals

Article

Temporal Microbial Dynamics in Feces Discriminate by Nutrition, Fecal Color, Consistency and Sample Type in Suckling and Newly Weaned Piglets

Barbara U. Metzler-Zebeli [1,2,*], Frederike Lerch [1,2], Fitra Yosi [1,2,3], Julia Vötterl [1,2], Juliane Ehmig [1,2], Simone Koger [2,4] and Doris Verhovsek [5]

1 Unit Nutritional Physiology, Department of Biomedical Sciences, University of Veterinary Medicine Vienna, 1210 Vienna, Austria
2 Christian-Doppler Laboratory for Innovative Gut Health Concepts of Livestock, University of Veterinary Medicine Vienna, 1210 Vienna, Austria
3 Department of Animal Science, Faculty of Agriculture, University of Sriwijaya, Palembang 30662, Indonesia
4 Institute of Animal Nutrition and Functional Plant Compounds, Department for Farm Animals and Veterinary Public Health, University of Veterinary Medicine Vienna, 1210 Vienna, Austria
5 University Clinic for Swine, Department for Farm Animals and Veterinary Public Health, University of Veterinary Medicine Vienna, 1210 Vienna, Austria
* Correspondence: barbara.metzler@vetmeduni.ac.at

Supplementary Materials

Citation: Metzler-Zebeli, B.U.; Lerch, F.; Yosi, F.; Vötterl, J.; Ehmig, J.; Koger, S.; Verhovsek, D. Temporal Microbial Dynamics in Feces Discriminate by Nutrition, Fecal Color, Consistency and Sample Type in Suckling and Newly Weaned Piglets. *Animals* **2023**, *13*, 2251. https://doi.org/10.3390/ani13142251

Academic Editor: Kelsy J. Robinson

Received: 6 June 2023
Revised: 5 July 2023
Accepted: 7 July 2023
Published: 9 July 2023

Copyright: © 2023 by the authors. Submitted for possible open access publication under the terms and conditions of the Creative Commons Attribution (CC BY) license (https://creativecommons.org/licenses/by/4.0/).

Table S1. Analyzed nutrient composition of the piglets and sow diet.

Chemical Composition, % DM	Lactation Diet[1]	Milk Replacer[2]	Prestarter Diet[3]
Dry matter, %	89.0	94.8	91.9
Crude ash	5.5	5.6	5.5
Crude protein	17.9	19.8	20.5
Crude fibre	5.8	0.4	5.2
Neutral-detergent fibre	17.3	3.0	15.2
Acid-detergent fibre	6.7	0.6	5.8
Acid-detergent lignin	1.8	0.3	1.5
Crude fat	5.2	7.8	7.5
Nitrogen-free extract	65.7	66.5	61.6
Starch	47.3	29.4	31.9
Sugar	5.5	26.8	14.2
Metabolizable energy, MJ/kg	14.7	16.9	15.3

[1]ZuchtsauenKorn S Vital, Garant-Tiernahrung GmbH, Pöchlarn, Austria. Ingredient composition: corn, wheat, barley, soybean meal, sunflower meal, wheat bran, apple pomace, soybean oil, calcium carbonate, monocalcium phosphate, sodium chloride, magnesium phosphate, fish oil, L-cellulose, molasses. Vitamin and mineral composition per kg feed: 10,000 IU of vitamin A, 1,800 IU of vitamin D, 100 mg of Fe as iron(II) sulfate, 15 mg of Cu as copper(II) sulfate, 90 mg of Zn as zinc sulfate, 40 mg of Mn as manganese(II) oxide, 1.5 mg of I as calcium iodate, 0.4 mg of Se as sodium selenite. Technological additives: 500 FTU phytase, 2 mg of butylated hydroxyanisole, 10 mg of butylated hydroxytoluene, 2 mg of propyl gallate.

[2]Weanplus-4, Startix, Voorthuizen, The Netherlands. Ingredient composition: whey powder, starch, soy protein, plant protein, plant oil (coconut oil and palm oil). Vitamin and mineral composition per kg feed: 25,000 IU of vitamin A, 8,000 IU of vitamin D3, 200 mg of vitamin E, 140 mg of Cu as chelate of glycine hydrate, 108 mg of Fe as chelate of glycine hydrate, 3 mg of I as calcium iodate, 115 mg of Zn as chelate of glycine hydrate, 35 mg of Mn as glycine chelate, 0.3 mg of Se as sodium selenite. Technological additives: 5 g of citric acid, 1 g of calcium formiate. Mixing ratio: 200 g of powder mixed into 1 L of 40°C warm water.

[3]Ferkelabsetzkorn OGT, Garant-Tiernahrung GmbH, Pöchlarn, Austria. Ingredient composition: oat flakes, barley, wheat, whey powder, soy protein concentrate, wheat bran, sucrose, soybean meal, soy oil, corn gluten, L-cellulose, monocalcium phosphate, fish oil, sodium chloride, magnesium phosphate, calcium carbonate and molasses. Vitamin and mineral composition per kg feed: 16,000 IU of vitamin A, 2,000 IU of vitamin D_3, 200 mg vitamin E, 120 mg Fe as iron(II) sulfate, 140 mg Cu as copper(II) sulfate, 120 mg Zn as zinc sulfate, 60 mg Mn as manganese(II) oxide, 1.5 mg I as calcium iodate, 0.5 mg Se as sodium selenite. Technological additives: 1,000 IU of phytase, 1,500 EPU of xylanase, 11 mg of beta hydroxy acid, 21 mg of butylated hydroxytoluene, 11 mg of propyl gallate.

animals

Table S2. Oligonucleotide primers for quantification of total bacterial 16S rRNA, protozoal 18S rRNA, fungal 26S rRNA and archaeal 16S rRNA gene copies.

Group		Primer sequence (5' to 3')[1]	Amplicon size (bp)	PCR Efficiency	Reference
Total bacteria	F:	CCTACGGGAGGCAGCAG	193	95.3%	[1]
	R:	ATTACCGCGGCTGCTGG			
Total protozoa	F:	GCTTTCGWTGGTAGTGTATT	233	99.6%	[2]
	R:	CTTGCCCTCYAATCGTWCT			
Total fungi and yeasts	F:	GCATATCAATAAGCGGAGGAAAAG	250	91.1%	[3]
	R:	ATTCCCAAACAACTCGACTC			
Total archaea	F:	CCGGAGATGGAACCTGAGAC	160	99.6%	[4]
	R:	CGGTCTTGCCCAGCTCTTATTC			

[1]F, forward primer; R, reverse primer.

References

1. Muyzer, G., De Waal, E.C., Uitterlinden, A.G. Profiling of complex microbial populations by denaturing gradient gel electrophoresis analysis of polymerase chain reaction-amplified genes coding for 16S rRNA. *Appl. Environ. Microbiol.* **1993**, *59*, 695–700.
2. Sylvester, J.T., Karnati, S. K. R., Yu, Z., Morrison, M., Firkins, J.L. Development of an assay to quantify rumen ciliate protozoal biomass in cows using real-time PCR. *J. Nutr.* **2004**, *134*, 3378–3384. doi:10.1093/jn/134.12.3378.
3. Urubschurov, V., Büsing, K., Janczyk, P., Souffrant, W.B., Zeyner, A. Development and evaluation of qPCR assay for quantitation of Kazachstania slooffiae and total yeasts occurring in the porcine gut. *Curr. Microbiol.* **2015**, *71*:373–381. doi:10.1007/s00284-015-0862-2.
4. Zhou, M., Hernandez-Sanabria, E., Guan, L.L. Assessment of the microbial ecology of ruminal methanogens in cattle with different feed efficiencies. *Appl. Environ. Microbiol.* **2009**, *75*, 6524–6533. doi:10.1128/AEM.02815-08.

animals

MDPI

Table S3. Descriptive statistics for average daily creep feed intake during the suckling period.*

Daily intake (g dry matter)	Mean	SE	Minimum	Maximum	Median
DoL 10-16	10	1.9	4	23	8
DoL 17-23	18	3.7	7	41	14
DoL 24-25	30	3.8	14	52	29
DoL 26-28	79	10.3	40	125	78

*Creep feed consumption was estimated at litter level. DoL, day of life; SE, standard error of the mean. Piglets in the creep-fed group were offered the milk replacer from DoL 10 to 23, transitioned from the milk replacer to the prestarter on DoL 24 and 25 and were offered the prestarter diet to 100% from DoL 26. Piglets in the sow milk only group received the prestarter from weaning on day 28 of life.

animals

Table S4. Body weight development of piglets selected for collection of feces.*

					P-value	
Item	Sow milk	Creep feed	SEM	litter_birth	diet	sex × diet
Birth weight	1.5	-	0.07	0.017	0.010	0.956
Day 2	1.6	-	0.09	0.036	0.016	0.852
Day 6	2.1	-	0.16	0.059	0.001	0.682
Day 13	4.4	3.4	0.23	0.239	0.013	0.566
Day 20	6.4	5.4	0.36	0.474	0.083	0.735
Day 27	8.6	7.1	0.47	0.172	0.067	0.742
Day 30	8.8	7.2	0.45	0.165	0.044	0.837
Day 34	8.9	7.2	0.45	0.39	0.030	0.909

*Values are least squares means ± standard error of the mean (SEM). Piglets were weaned on day 28 of life.

animals

MDPI

Table S5. Age-related development of relative bacterial abundances (% of total reads) in feces from suckling and newly weaned piglets fed either only sow milk or additional creep feed from day 10 of life.*

Day of Life (DoL)	2	6	13		20		27		30		34					
Feeding (Feed)	Sow Milk	Sow Milk	Sow Milk	Creep feed	Sow Milk	Creep feed	Sow Milk	Creep feed	Sow Milk	Creep feed	Sow Milk	Creep feed	Pooled SEM	DoL	Feed	DoL × Feed
Lactobacillus	1.91	4.82	7.93	9.15	4.18	4.56	2.79	4.04	1.57	1.90	5.96	4.14	1.517	<0.001	0.326	0.292
Escherichia_Shigella	38.11	24.91	22.52	27.49	9.64	12.99	6.34	6.01	7.40	4.99	2.12	2.52	3.299	<0.001	0.516	0.912
Bacteroides	11.39	20.09	11.88	13.73	7.73	6.21	5.83	8.93	3.95	5.26	1.46	0.55	2.159	<0.001	0.067	0.240
Prevotella	0.80	1.90	3.36	1.66	3.60	2.71	4.47	6.11	3.68	9.61	6.59	7.51	1.141	<0.001	0.387	0.012
Actinobacillus	3.99	1.67	0.44	0.66	0.55	0.12	0.18	1.44	0.16	0.58	0.08	0.04	0.833	<0.001	0.332	0.595
Fusobacterium	13.65	9.32	1.27	0.67	0.82	2.95	5.08	8.37	1.84	3.12	0.88	0.06	1.881	<0.001	0.809	0.374
Alloprevotella	0.28	0.92	0.65	0.40	1.12	1.32	1.82	3.65	2.09	5.88	3.00	4.08	0.699	<0.001	0.004	0.067
Rikenellaceae_RC9_gut_group	0.09	0.25	2.42	1.47	7.54	4.91	9.30	4.63	4.25	2.17	3.01	4.57	1.039	<0.001	0.026	0.072
Clostridium_sensu_stricto_1	13.47	3.10	1.55	2.11	1.69	1.43	2.52	1.33	2.94	1.73	1.30	1.47	1.635	<0.001	0.477	0.959
Streptococcus	2.97	2.26	1.95	0.88	0.77	0.45	1.47	1.75	2.48	1.22	0.20	0.08	0.676	<0.001	0.524	0.706
Lachnoclostridium	0.79	6.61	6.11	5.21	4.76	3.78	5.28	4.17	2.60	2.93	0.68	0.52	1.099	<0.001	0.827	0.199
Phascolarctobacterium	0.07	0.31	1.44	0.64	2.42	2.02	2.28	3.08	2.73	3.75	2.89	3.75	0.424	<0.001	0.543	0.165
Campylobacter	0.94	0.81	1.45	1.39	0.80	1.36	0.99	1.49	3.20	2.91	4.00	2.02	0.763	0.006	0.667	0.432
Megasphaera	0.07	0.14	0.18	0.48	0.82	1.15	1.82	2.75	2.16	1.92	4.46	3.52	0.879	<0.001	0.954	0.971
Christensenellaceae_R_7_group	0.04	0.18	0.73	1.15	7.30	6.14	4.18	2.74	4.03	1.36	4.27	2.87	0.861	<0.001	0.044	0.560
Prevotellaceae_NK3B31_group	0.03	0.34	0.67	0.10	0.61	0.92	0.84	1.66	1.98	2.71	3.03	2.57	0.406	<0.001	0.705	0.463
Oscillospiraceae_UCG_002	0.03	0.89	2.10	2.59	5.29	2.05	2.37	1.75	2.74	2.14	2.95	3.13	0.540	<0.001	0.044	0.018
Roseburia	0.14	0.16	0.43	0.32	0.77	1.50	1.17	0.99	1.99	1.99	1.93	1.69	0.402	<0.001	0.870	0.927
NK4A214_group	0.02	0.21	1.33	1.19	2.46	1.57	1.45	0.82	2.09	1.51	2.27	2.50	0.312	<0.001	0.134	0.481
Moraxella	0.22	0.05	0.03	0.04	0.01	0.02	0	0	0.01	0.01	0	0	0.047	<0.001	0.056	0.002
Veillonella	1.09	1.00	0.22	0.46	0.17	0.21	0.16	0.24	0.04	0.09	0.06	0	0.149	<0.001	0.883	0.717
Enterococcus	0.68	2.33	3.67	3.18	0.73	1.66	1.03	0.78	0.07	0.05	0.08	0.12	0.585	<0.001	0.972	0.919

**Day of Life (DoL)**	**2**	**6**	**13**		**20**		**27**		**30**		**34**					
**Feeding (Feed)**	**Sow Milk**	**Sow Milk**	**Sow Milk**	**Creep feed**	**Sow Milk**	**Creep feed**	**Sow Milk**	**Creep feed**	**Sow Milk**	**Creep feed**	**Sow Milk**	**Creep feed**	**Pooled SEM**	**DoL**	**Feed**	**DoL × Feed**
Family_XIII_AD3011_group	0.01	0.04	0.44	0.06	1.29	0.93	1.31	0.85	1.46	1.59	1.70	1.85	0.253	<0.001	0.325	0.779
Desulfovibrio	0.08	0.63	0.83	0.62	0.97	0.77	1.26	1.03	0.80	0.83	0.66	0.63	0.149	<0.001	0.154	0.867
Lachnospiraceae_NK4A136_group	0	0.01	0.01	0.01	0.42	0.31	0.16	0.98	1.94	3.19	1.41	1.82	0.345	<0.001	0.072	0.344
Colidextribacter	0.06	0.41	0.34	0.65	0.68	0.69	0.80	0.58	0.65	0.66	0.59	0.81	0.137	<0.001	0.833	0.424
Ruminococcus	0.08	0.59	0.54	0.78	0.86	1.05	1.33	1.30	1.46	1.50	0.80	1.24	0.242	<0.001	0.567	0.858
Rothia	0.08	0.07	0.04	0.05	0.04	0.04	0.01	0.01	0.01	0	0	0	0.016	<0.001	0.205	0.128
Subdoligranulum	0.02	0.11	0.15	0.55	0.98	0.32	0.69	1.47	0.57	1.61	1.23	1.60	0.274	<0.001	0.078	0.045
Mitsuokella	0.06	0.02	0.02	0.01	0.23	0.14	0.64	0.63	0.51	0.67	3.74	1.63	0.579	<0.001	0.364	0.453
Oscillibacter	0.01	0.02	0.23	0.06	0.98	0.88	1.20	0.89	0.72	0.90	0.51	0.61	0.209	<0.001	0.749	0.915
Butyricimonas	0.04	0.48	1.85	0.79	0.91	0.41	0.71	0.61	0.35	0.21	0.05	0.02	0.221	<0.001	0.027	0.164
Coprococcus	0.05	0.08	0.07	0.30	0.80	0.32	1.19	0.39	1.40	1.10	1.46	3.03	0.323	<0.001	0.900	0.016
Parabacteroides	0.30	0.80	1.28	0.25	0.58	0.82	0.28	0.39	0.81	0.41	0.78	0.41	0.261	0.315	0.013	0.096
Helicobacter	0.03	0.10	0.15	0.24	0.27	2.90	0.39	0.63	1.32	1.49	0.61	0.38	0.524	0.006	0.200	0.086
Agathobacter	0	0.02	0.04	0.00	0	0	0	0.24	0.70	1.01	1.34	2.23	0.233	<0.001	0.221	0.333
Terrisporobacter	0.39	0.10	0.32	0.25	0.14	0.74	0.20	0.23	0.66	0.95	0.44	0.59	0.177	0.004	0.293	0.220
Prevotellaceae_UCG_001	0	0.01	0.64	0.14	0.28	0.88	0.49	0.74	0.61	0.47	0.36	0.59	0.323	0.157	0.796	0.655
Tuzzerella	0.08	0.21	0.23	0.15	0.37	1.27	0.46	0.57	0.32	0.56	0.83	0.89	0.259	0.007	0.308	0.468
Romboutsia	0.19	0.08	0.32	0.32	0.29	0.30	0.23	0.39	0.41	0.21	0.03	0.03	0.112	0.040	0.407	0.408
Blautia	0.01	0.08	0.05	0.05	0.31	0.21	0.29	0.61	0.76	1.18	1.87	2.54	0.255	<0.001	0.229	0.644
Dorea	0.04	0.08	0.24	0.34	1.10	0.60	0.43	0.63	0.62	0.66	0.68	0.94	0.157	<0.001	0.922	0.274
Porphyromonas	0.05	0.32	0.47	0.17	0	0.02	0.18	0.00	0.36	0.06	0.05	0.01	0.158	0.152	0.133	0.822
Alistipes	0.01	0.33	0.90	0.91	0.71	0.15	0.79	0.82	0.32	0.30	0.01	0.05	0.188	<0.001	0.472	0.633
Lachnospiraceae_AC2044_group	0	0.01	0.03	0.07	0.39	0.63	0.08	0.04	0.50	1.61	0.64	0.99	0.200	<0.001	0.033	0.054

*Values are least squares means ± pooled standard error of the mean (SEM). Piglets were weaned on day 28 of life. Proportional abundances are presented that represent >0.2% of all reads.

animals

MDPI

Table S6. Differences in relative bacterial abundances (% of total reads) in feces of different colors and consistencies obtained from suckling and newly weaned piglets during the suckling and early postweaning phase.*

**Day of Life (DoL)**	**2**	**2**	**2**	**2**	**2**	**6**	**6**	**6**	**13**	**13**	**13**	**13**	**20**	**20**	**20**	**20**	**20**	**20**	**20**	**20**
**Color**	**brown**	**brown**	**yellow**	**yellow**	**yellow**	**brown**	**yellow**	**yellow**	**brown**	**brown**	**yellow**	**yellow**	**brown**	**brown**	**brown**	**grey**	**grey**	**yellow**	**yellow**	**yellow**
**Consistency**	**balls**	**meconium**	**balls**	**very soft**	**soft**	**balls**	**balls**	**soft**	**balls**	**very soft**	**balls**	**soft**	**balls**	**very soft**	**soft**	**balls**	**soft**	**balls**	**very soft**	**soft**
Lactobacillus	0	0	2.94	3.29	3.46	0	5.86	5.10	11.47	0.58	7.99	0	4.92	0.25	0	5.14	0	4.83	5.70	2.44
Escherichia_Shigella	43.84	59.35	34.00	18.67	23.54	27.82	24.84	10.97	23.75	11.65	26.69	6.68	15.93	5.62	4.04	5.18	5.51	12.79	2.19	7.70
Bacteroides	11.91	8.40	11.41	5.72	21.10	14.60	20.73	26.50	16.56	0.80	11.95	6.49	2.54	3.65	14.33	2.59	2.86	7.45	13.70	6.74
Prevotella	0.33	0.46	1.14	0.24	0.17	3.88	1.54	2.39	1.58	26.89	1.80	7.64	8.14	0.45	1.12	5.89	2.03	1.73	0.75	8.01
Actinobacillus	1.58	1.08	4.33	9.25	8.77	0.85	1.80	2.91	0.31	0	0.70	0.71	0.77	0.97	0.33	0.72	0.68	0.20	0.39	0.06
Fusobacterium	9.62	7.22	15.30	7.42	26.81	9.40	8.96	21.15	1.49	0	0.97	0	0.25	0	0	0	0	0.87	18.89	0.70
Alloprevotella	0.43	0.26	0.30	0	0.19	2.14	0.70	0.65	0.42	4.29	0.38	1.99	0.92	7.92	0.38	5.33	0.72	0.88	0.55	2.03
Rikenellaceae_RC9_gut_group	0	0	0.61	2.02	0	0	0.50	0.87	1.56	6.44	1.35	17.78	7.40	1.09	12.55	11.37	0.99	5.74	2.22	11.43
Clostridium_sensu_stricto_1	23.32	3.61	12.41	47.72	5	9.49	2.05	0	2.43	0.64	1.67	0.87	3.02	1.29	3.22	1.13	1.26	1.43	0.42	0.57
Streptococcus	3.24	0.62	4.05	0.75	1.13	2.73	2.12	3.88	1.62	0	1.43	0.29	1.51	0.88	0.43	0.67	0.76	0.41	0.61	0.34
Lachnoclostridium	0.16	0	1.04	0.80	1.79	4.74	6.90	5.92	5.31	4.30	5.89	4.68	3.73	0	5.01	2.21	1.00	4.88	2.83	5.02
Phascolarctobacterium	0	0	0.13	0.32	0	0.02	0.36	0.30	0.69	3.96	1.06	1.36	2.40	3.83	1.99	2.40	2.08	2.01	2.50	2.81
Campylobacter	0	0	1.71	0.29	0.25	0	0.98	0.64	1.42	0	1.41	3.41	0.74	0.51	0.87	0.23	0.0	1.24	1.54	0.89
Megasphaera	0.08	0.27	0.02	0	0.11	0.56	0.07	0	0.07	0.15	0.44	0.38	3.34	0.44	0.11	0.18	0.23	0.67	0.12	0.63
Christensenellaceae_R_7_group	0	0	0.29	0.58	0	0	0.21	0.55	0.88	0.22	1.03	0	3.10	11.77	10.43	12.48	25.37	7.25	2.21	3.18
Prevotellaceae_NK3B31_group	0.01	0	0.11	0.13	0	0.03	0.40	0.35	0.16	9.38	0.15	0.10	0.24	1.96	0	2.34	6.50	0.59	0	1.62
UCG_002	0	0	0.11	0.24	0	0.82	0.88	1.90	2.29	1.08	2.49	0.42	2.06	1.71	11.08	2.52	0.88	3.95	2.12	5.63
Roseburia	0.01	0	0.21	0	0.29	0.02	0.17	0.47	0.70	0.06	0.26	0.09	0.29	1.03	0	0.58	0.36	1.07	5.04	0.33
NK4A214_group	0	0	0.02	0	0.10	0.17	0.23	0	1.51	1.76	1.14	1.20	1.54	5.14	2.72	1.00	2.49	2.07	1.07	2.28
Moraxella	0.17	0.07	0.12	1.47	0.57	0	0.06	0.04	0.02	0	0.04	0	0.01	0	0	0	0	0.02	0	0
Veillonella	1.51	0.65	1.10	0.77	1.52	1.55	0.91	0.75	0.24	0.87	0.36	0.43	0.08	0.23	0.06	0.23	0.07	0.18	0.44	0.29

**Day of Life (DoL)**	**2**	**2**	**2**	**2**	**2**	**6**	**6**	**6**	**13**	**13**	**13**	**13**	**20**	**20**	**20**	**20**	**20**	**20**	**20**	**20**
**Color**	**brown**	**brown**	**yellow**	**yellow**	**yellow**	**brown**	**yellow**	**yellow**	**brown**	**brown**	**yellow**	**yellow**	**brown**	**brown**	**brown**	**grey**	**grey**	**yellow**	**yellow**	**yellow**
**Consistency**	**balls**	**meconium**	**balls**	**very soft**	**soft**	**balls**	**balls**	**soft**	**balls**	**very soft**	**balls**	**soft**	**balls**	**very soft**	**soft**	**balls**	**soft**	**balls**	**very soft**	**soft**
Enterococcus	1.25	1.89	0.33	0	0.49	3.39	2.23	0	3.07	1.03	3.76	0.75	1.01	1.11	0.79	0.97	1.09	1.47	0.32	0.48
Family_XIII_AD3011_group	0	0.01	0.02	0	0	0.02	0.04	0.02	0.04	1.58	0.19	2.90	0.33	8.79	0.43	1.86	1.50	0.94	0.23	2.13
Desulfovibrio	0	0.0	0.16	0.08	0	0.83	0.62	0.07	0.79	1.46	0.62	2.10	0.41	0.95	2.39	0.90	0.67	0.73	1.60	1.77
Lachnospiraceae_NK4A136_group	0	0.0	0.06	0.15	0	0	0.03	0.13	0	0	0.04	0	0.06	3.21	0	1.45	0.83	0.32	0	0.39
Colidextribacter	0.02	0.02	0.10	0	0.02	0.46	0.37	1.51	0.59	1.49	0.43	0.08	0.28	0.42	0.28	1.26	0.87	0.72	1.15	0.71
Ruminococcus	0	0	0.16	0.15	0	0.45	0.55	3.04	0.39	0.34	0.79	0.30	0.71	0.58	2.52	1.41	1.01	1.01	0.39	0.99
Rothia	0.08	0.06	0.08	0.18	0.06	0.13	0.06	0.01	0.05	0.04	0.05	0	0.04	0.04	0.04	0.02	0.07	0.04	0.02	0.06
Subdoligranulum	0.03	0.04	0.01	0.01	0.02	0.12	0.12	0	0.28	0.17	0.34	1.37	0.19	0.13	5.17	0.46	0.81	0.71	0.09	0.41
Mitsuokella	0.13	0.25	0	0.08	0.04	0.09	0.01	0	0.04	0.06	0.02	0	0.35	0	0.05	0.17	0.11	0.19	0.02	0.19
Oscillibacter	0	0.08	0	0	0.01	0.09	0.02	0	0.10	1.46	0.08	1.02	0.12	0.78	0.20	1.83	5.54	0.68	0.16	3.79
Butyricimonas	0	0	0.10	0.11	0	1.25	0.34	0.55	0.77	1.13	1.20	10.74	0.11	0	2.49	0	0.13	0.81	0.27	0.96
Coprococcus	0	0	0.08	0.05	0	0	0.10	0.04	0.19	0	0.20	0	0.45	0.81	1.29	0	1.79	0.60	0.11	0.37
Parabacteroides	0.20	0.40	0.33	0	0.23	0.79	0.80	1.05	0.54	0.05	0.91	0.09	0.04	0.20	0.09	7.03	7.27	0.47	0.19	0.46
Helicobacter	0.04	0.05	0.04	0	0.07	0	0.13	0.03	0.06	0.14	0.20	1.54	0.04	0.46	0.03	0.13	0.30	2.41	0.22	1.22
Agathobacter	0	0.05	0.01	0.07	0.03	0	0.06	0.05	0.05	0	0.02	0	0.03	0	0.05	0.0	0.04	0.00	0.08	0
Terrisporobacter	0.23	0.17	0.35	2.28	0.18	0.21	0.09	0	0.19	0.13	0.33	0.13	1.35	0.39	0.12	0.15	0.68	0.31	0.11	0.06
Prevotellaceae_UCG_001	0.25	0.18	0	0.12	0	0.23	0	0.02	1.29	0	0.05	0.20	0.25	0.22	0.48	0.16	0.23	0.79	0.13	0.39
Tuzzerella	0	0	0.22	0.04	0	0.07	0.22	0.80	0.18	0.30	0.18	0.15	0.59	0.32	0	0.10	0.29	0.24	6.61	1.04
Romboutsia	0.10	1.01	0	0	0.04	0.07	0.09	0	0.32	0.11	0.34	0.10	0.55	0.35	0.30	0.19	0.70	0.24	0.08	0.32
Blautia	0.09	0.07	0	0	0.03	0.27	0.05	0	0.11	0.09	0.03	0.09	0.46	0.09	0.46	0.09	0.09	0.28	0.05	0.03
Dorea	0.0	0.0	0.09	0	0.02	0.02	0.10	0	0.21	0.59	0.30	0.84	0.43	0	0.69	0.02	0.97	0.98	0.37	1.70
Porphyromonas	0.08	0.06	0.05	0	0.11	0.07	0.41	0	0.10	0.10	0.42	0.06	0.02	0.12	0.06	0	0	0	0.07	0.06
Alistipes	0	0	0.09	0.28	0.06	0.37	0.32	0.46	0.80	0.23	1.00	0.25	0.23	0.13	1.01	0.0	0.58	0.46	0	1.26
Lachnospiraceae_AC2044_group	0	0.02	0.01	0.00	0.03	0.03	0.01	0	0	0.23	0.06	0.11	0.12	1.35	0.06	1.39	0.46	0.66	0	0.31

Day of Life (DoL)	27	27	27	27	27	27	27	30	30	30	30	30	34	34	34	34	34	
**Color**	**brown**	**brown**	**brown**	**grey**	**yellow**	**yellow**	**yellow**	**brown**	**brown**	**brown**	**yellow**	**yellow**	**brown**	**brown**	**brown**	**yellow**	**yellow**	**Pooled**
**Consistency**	**balls**	**diarrhea**	**soft**	**soft**	**balls**	**diarrhea**	**soft**	**balls**	**diarrhea**	**soft**	**balls**	**soft**	**balls**	**diarrhea**	**soft**	**balls**	**soft**	**SEM**
Lactobacillus	1.73	4.01	3.49	0.32	3.49	5.50	3.40	1.96	0.65	1.18	2.70	1.59	2.92	9.79	2.20	1.07	6.11	3.960
Escherichia_Shigella	8.48	4.11	1.90	8.38	6.58	2.81	8.10	4.55	9.68	0	8.78	5.51	0	3.63	7.19	3.59	13.66	8.029
Bacteroides	5.02	6.61	11.85	10.21	8.07	2.76	8.06	2.63	0.74	2.42	11.72	3.16	1.61	1.03	0.00	11.38	0	5.547
Prevotella	1.96	11.69	5.67	29.05	2.28	11.10	5.87	8.01	6.38	15.96	2.67	6.28	7.20	5.98	9.16	0.19	10.13	2.505
Actinobacillus	0.62	1.40	1.41	0.61	0.87	0.45	0.49	0.11	0.70	0.30	0.37	0.62	0.12	0	0.22	0.55	0	2.095
Fusobacterium	1.21	5.25	0.10	0	7.69	18.70	5.66	1.26	0.48	2.04	6.16	2.46	1.28	0.29	0.00	3.48	0	4.641
Alloprevotella	1.18	11.06	3.59	6.14	1.89	3.12	1.16	3.03	6.93	0.83	3.17	3.53	2.89	4.62	3.28	3.67	2.24	1.735
Rikenellaceae_RC9_gut_group	13.09	2.89	6.08	6.65	6.84	2.71	7.61	3.45	1.97	4.85	3.70	3.17	2.79	3.81	5.39	3.36	6.78	2.670
Clostridium_sensu_stricto_1	5.84	0.82	1.72	0.40	2.01	0.34	0.40	2.71	1.72	2.52	2.50	1.44	1.43	1.06	2.29	0.00	0.72	3.848
Streptococcus	0.83	0.51	0.63	0.47	2.90	0.50	0.36	1.77	1.64	0.50	2.66	0.38	0.19	0	0.42	0.18	0	1.747
Lachnoclostridium	4.38	0.99	1.48	2.10	4.15	1.28	12.97	1.92	1.52	1.14	5.49	3.19	0.92	0.47	0.12	0.58	0.78	2.767
Phascolarctobacterium	3.29	3.07	8.13	5.69	1.36	3.13	2.40	2.32	5.50	3.87	1.91	4.03	3.05	3.78	3.74	1.59	0.56	1.008
Campylobacter	1.25	1.54	0.72	0.61	1.65	0.40	0.76	5.34	1.35	0.69	2.72	0.58	2.71	1.86	1.05	37.59	0.34	1.543
Megasphaera	0.91	3.04	4.92	3.63	0.85	4.20	4.54	1.95	3.65	0.29	1.18	0.36	1.36	7.20	4.99	0	0.26	2.202
Christensenellaceae_R_7_group	5.73	0	3.90	0.00	4.33	1.28	2.76	2.87	1.00	3.37	3.73	5.42	4.07	3.43	3.16	2.16	0.27	2.144
Prevotellaceae_NK3B31_group	1.83	6.03	2.14	6.22	0.30	0.67	0.34	2.56	2.80	5.14	0.72	3.74	2.53	2.17	4.43	0.13	4.97	0.873
UCG_002	3.74	0.32	1.16	0.29	1.88	4.34	1.31	1.55	2.60	4.30	3.00	1.35	2.99	2.65	3.59	0.46	6.92	1.487
Roseburia	0.61	4.03	2.64	0.46	0.38	0.79	1.61	1.66	3.78	0.93	0.89	2.09	1.68	1.87	2.14	0.30	2.03	0.981
NK4A214_group	1.26	0.78	0.54	0.32	1.21	0.83	1.61	2.16	1.90	2.13	0.82	5.46	2.92	2.22	1.83	0.38	2.54	0.798
Moraxella	0.01	0	0.02	0	0.01	0	0	0	0	0.01	0.02	0.02	0.01	0.01	0	0.01	0	0.109
Veillonella	0.04	0.70	0.47	0.34	0.07	0.31	0.23	0.01	0.17	0.03	0.03	0.05	0	0.08	0.01	0.05	0.02	0.386
Enterococcus	0	1.03	0.41	1.11	1.62	0.0	0.49	0.19	1.04	0	0	0	0	0.12	1.06	0	1.03	1.519
Family_XIII_AD3011_group	1.58	2.00	0.63	0.34	0.94	1.35	0.78	1.43	1.72	0.77	1.42	4.12	2.16	1.73	0.67	4.88	1.82	0.563
Desulfovibrio	1.23	0.93	1.27	1.58	0.94	1.17	1.66	0.50	0.75	1.22	1.14	1.29	0.67	0.42	0.81	1.28	1.01	0.377
Lachnospiraceae_NK4A136_group	0.18	0.17	4.33	0	0.49	0.02	0	2.25	3.04	8.38	0.58	6.29	1.59	1.08	2.78	1.02	0.30	0.745

**Day of Life (DoL)**	**27**	**27**	**27**	**27**	**27**	**27**	**27**	**30**	**30**	**30**	**30**	**30**	**34**	**34**	**34**	**34**	**34**	
**Color**	**brown**	**brown**	**brown**	**grey**	**yellow**	**yellow**	**yellow**	**brown**	**brown**	**brown**	**yellow**	**yellow**	**brown**	**brown**	**brown**	**yellow**	**yellow**	**Pooled**
**Consistency**	**balls**	**diarrhea**	**soft**	**soft**	**balls**	**diarrhea**	**soft**	**balls**	**diarrhea**	**soft**	**balls**	**soft**	**balls**	**diarrhea**	**soft**	**balls**	**soft**	**SEM**
Colidextribacter	0.68	0.85	0.93	0.18	0.74	1.21	0.11	0.58	0.63	0.69	0.60	2.25	0.87	0.77	0.32	0.26	0.77	0.350
Ruminococcus	3.92	0.24	2.34	0.09	1.32	0.04	0.18	1.72	0.82	2.21	1.55	2.16	1.26	0.59	1.39	0	0.94	0.571
Rothia	0.01	0	0.02	0	0.02	0.00	0.01	0.01	0	0.01	0.02	0.01	0.01	0	0	0.02	0	0.042
Subdoligranulum	1.60	0.51	1.54	0.14	1.17	0.55	0.94	1.26	0.59	4.10	0.64	0.13	1.66	1.45	1.21	0.14	0.66	0.700
Mitsuokella	0.24	1.48	0.97	0.19	0.23	1.49	1.09	0.31	1.35	1.19	0.04	0.44	2.06	3.89	2.69	0	0.06	1.549
Oscillibacter	2.07	1.92	0.48	1.48	0.94	1.10	0.25	0.88	0.79	1.35	0.57	1.06	0.38	0.59	0.93	0.32	0.43	0.466
Butyricimonas	0.52	0.24	0.20	0.92	0.62	0.59	1.33	0.21	0	0.13	0.68	0.42	0.07	0.06	0	0.22	0.05	0.469
Coprococcus	1.08	0.07	0.54	0.12	1.20	0.25	0.26	1.51	0.96	2.30	0.75	3.14	4.34	0.92	0.74	0.50	0.18	0.773
Parabacteroides	0.42	1.16	0.89	0.25	0.13	0.57	0.03	0.52	0.72	0.13	0.61	1.97	0.51	0.55	0.72	1.33	0.18	0.636
Helicobacter	0.88	0.23	0.10	0.56	0.70	0.03	0.27	2.03	0.19	0.11	2.26	0.43	1.01	0.10	0.02	0.87	0.02	1.446
Agathobacter	0	0	0.67	0	0.16	0	0.07	0.71	1.94	0.67	0.07	0.07	1.89	1.85	2.00	0.07	0	0.590
Terrisporobacter	0.20	0.16	0.16	0.26	0.30	0.14	0.05	1.15	0.34	1.94	0.60	0	0.63	0.12	0.93	0.76	0.13	0.441
Prevotellaceae_UCG_001	0.35	0.46	0.31	0.28	0.22	0.54	2.36	0.68	0.73	0.51	0.26	0	0.49	0.39	0.60	0.72	0.32	0.805
Tuzzerella	0.38	0.69	1.50	0.01	0.32	1.27	0.23	0.29	0.86	0.32	0.17	1.15	1.09	1.05	0	1.60	1.71	0.568
Romboutsia	0.47	0.11	0.60	0.10	0.38	0.05	0.14	0.37	0.18	0.16	0.44	0	0	0	0.12	0.30	0.11	0.288
Blautia	1.00	0.12	0.18	0.20	0.59	0.12	0.13	1.53	0.42	1.82	0.63	0.23	3.92	1.42	0.64	0	0.42	0.589
Dorea	0.38	0.31	0.26	0.12	0.43	0.47	1.29	0.77	0.29	1.13	0.67	0.75	1.11	0.82	0.35	0	0.63	0.398
Porphyromonas	0	0.02	0.06	0.10	0.20	0.05	0.00	0.21	0.08	0.01	0.42	0.10	0.05	0	0.08	0.05	0.10	0.420
Alistipes	1.05	0	1.88	1.29	0.48	0.16	1.83	0.16	0.29	0.25	0.58	0	0.15	0	0	0	0	0.470
Lachnospiraceae_AC2044_group	0.13	0.09	0.03	0	0.06	0.11	0	0.77	1.59	3.56	0.22	0.83	0.71	0.75	1.19	0.82	0.66	0.505

	P-value						
	**DoL**	**Color**	**DoL × Color**	**Consistency**	**DoL × Consistency**	**Color × Consistency**	**DoL × Color × Consistency**
Lactobacillus	0.900	0.516	0.431	0.813	0.398	0.931	0.951
Escherichia_Shigella	0.0002	0.887	0.846	0.069	0.752	0.875	0.998
Bacteroides	0.036	0.826	0.274	0.267	0.473	0.477	0.915
Prevotella	<0.001	0.003	0.0002	0.072	<0.001	0.403	0.116
Actinobacillus	0.003	0.998	0.971	0.774	0.887	0.989	0.997
Fusobacterium	0.004	0.037	0.875	0.800	0.044	0.210	0.855
Alloprevotella	0.182	0.015	0.847	0.013	0.238	0.008	0.714
Rikenellaceae_RC9_gut_group	<0.001	0.926	0.462	0.179	0.007	0.110	0.645
Clostridium_sensu_stricto_1	<0.001	0.329	0.289	<0.001	<0.001	0.986	0.994
Streptococcus	0.608	0.995	0.992	0.568	0.986	0.975	0.941
Lachnoclostridium	0.177	0.164	0.976	0.440	0.938	0.827	0.242
Phascolarctobacterium	<0.001	0.273	0.211	0.110	0.080	0.740	0.136
Campylobacter	<0.001	0.070	<0.001	0.015	<0.001	<0.001	<0.001
Megasphaera	0.509	0.799	0.985	0.742	0.541	0.974	0.933
Christensenellaceae_R_7_group	<0.001	0.032	0.006	0.198	0.697	0.080	0.019
Prevotellaceae_NK3B31_group	0.021	<0.001	0.039	0.0001	<0.001	0.016	0.758
UCG_002	0.017	0.413	0.761	0.725	0.101	0.126	0.018
Roseburia	0.115	0.824	0.251	0.087	0.192	0.936	0.154
NK4A214_group	<0.001	0.612	0.469	0.818	0.351	0.039	0.028
Moraxella	<0.001	0.972	0.997	<0.001	<0.001	0.999	1.000
Veillonella	<0.001	0.837	0.531	0.147	0.986	0.989	0.985
Enterococcus	0.769	0.861	0.925	0.918	0.957	0.900	0.933
Family_XIII_AD3011_group	<0.001	0.172	0.103	0.014	<0.001	<0.001	<0.001
Desulfovibrio	<0.001	0.626	0.475	0.774	0.078	0.275	0.510

	P-value						
	**DoL**	**Color**	**DoL × Color**	**Consistency**	**DoL × Consistency**	**Color × Consistency**	**DoL × Color × Consistency**
Lachnospiraceae_NK4A136_group	<0.001	0.004	0.700	0.015	<0.001	0.106	0.014
Colidextribacter	0.003	0.193	0.362	0.188	0.303	0.352	0.078
Ruminococcus	0.004	0.417	0.010	0.043	0.001	0.339	0.238
Rothia	0.032	0.937	0.671	0.923	0.920	0.980	0.995
Subdoligranulum	0.602	0.019	0.591	0.032	0.090	0.043	0.027
Mitsuokella	0.912	0.812	0.990	0.916	1.000	1.000	1.000
Oscillibacter	0.002	0.004	0.027	0.083	0.0010	0.032	0.074
Butyricimonas	<0.001	0.602	0.068	0.002	<0.001	0.759	0.142
Coprococcus	0.043	0.718	0.436	0.476	0.271	0.686	0.394
Parabacteroides	0.037	<0.001	0.005	0.989	0.975	0.993	0.604
Helicobacter	0.969	0.967	0.993	0.823	0.998	0.942	0.970
Agathobacter	0.167	0.476	0.532	0.968	0.769	0.968	0.972
Terrisporobacter	0.212	0.247	0.357	0.934	0.104	0.536	0.381
Prevotellaceae_UCG_001	0.856	0.731	0.475	0.930	0.910	0.956	0.623
Tuzzerella	<0.001	<0.001	0.006	<0.001	<0.001	<0.001	0.002
Romboutsia	0.635	0.780	0.969	0.027	0.999	0.811	0.913
Blautia	0.195	0.225	0.106	0.281	0.915	0.599	0.211
Dorea	0.046	0.290	0.549	0.294	0.502	0.465	0.709
Porphyromonas	0.986	0.935	0.992	0.916	0.996	0.996	0.998
Alistipes	0.025	0.503	0.992	0.175	0.091	0.996	0.732
Lachnospiraceae_AC2044_group	0.022	0.072	0.267	0.840	0.487	0.193	0.291

*Values are least squares means ± pooled standard error of the mean (SEM). Piglets were weaned on day 28 of life. Proportional abundances are presented that represent >0.2% of all reads.

animals

MDPI

Table S7. Differences in relative bacterial abundances (% of total reads) in different fecal sample types obtained from suckling and newly weaned piglets during the suckling and early postweaning phase.*

Day of Life (DoL)	2				6			13			20			27			30			34			P-value			
Sample Type	F + S	F	M	S	F + S	F	S	F + S	F	S	F + S	F	S	F + S	F	S	F + S	F	S	F + S	F	S	Pooled SEM	DoL	Type	DoL × type
Lactobacillus	0.89	1.10	0.02	2.93	6.31	3.75	4.13	11.58	14.89	2.25	2.24	4.95	4.30	2.22	3.62	3.46	0.85	2.83	1.42	1.64	8.11	2.43	2.234	<0.001	0.239	0.004
Escherichia_Shigella	34.18	24.44	57.97	34.21	16.18	20.12	33.07	11.92	21.47	33.57	7.80	8.85	15.76	2.10	3.41	11.13	5.55	5.11	6.83	3.00	4.64	0	4.836	<0.001	0.0001	0.107
Bacteroides	15.67	6.33	9.64	10.47	28.47	20.92	13.22	16.04	12.47	11.63	9.47	6.55	6.69	4.37	7.68	7.77	0.63	4.17	5.64	0	0.71	1.54	3.263	<0.001	0.601	0.100
Prevotella	0.56	0	0.53	1.03	3.15	4.39	0.25	7.85	2.06	0.49	2.83	4.08	2.03	16.21	5.70	1.81	8.22	6.31	6.47	7.83	7.57	6.47	1.707	<0.001	0.002	0.005
Actinobacillus	4.13	18.33	1.11	4.20	0.96	1.02	2.45	0.54	0.90	0.28	0.73	0.48	0.02	0.26	0.46	1.46	0.99	0.21	0.32	0.19	0.11	0	1.258	<0.001	0.0006	0.145
Fusobacterium	18.48	9.07	7.26	13.90	6.21	16.20	9.44	0	1.23	1.44	4.34	1.88	1.07	1.78	5.86	9.25	2.23	0.32	3.53	0	0	1.58	2.887	<0.001	0.291	0.343
Alloprevotella	0.16	0.14	0.10	0.38	1.05	0.09	1.09	1.19	0.25	0.45	1.80	1.41	0.77	5.32	2.66	2.15	9.46	3.27	3.18	4.08	3.99	2.96	1.072	<0.001	0.055	0.149
Rikenellaceae_RC9_gut_group	0	0.89	0.0	0.58	0	0	0.91	4.48	1.77	0.95	2.75	8.99	3.70	9.29	9.07	3.40	3.35	3.73	2.94	7.01	4.51	2.49	1.586	<0.001	0.132	0.019
Clostridium_sensu_stricto_1	16.19	30.37	3.63	14.66	3.28	5.44	2.32	1.02	1.91	2.13	0.72	2.14	1.06	0.33	0.89	3.81	1.35	1.20	3.06	2.29	1.28	1.28	2.541	<0.001	0.0003	0.785
Streptococcus	2.18	1.07	0.69	4.05	2.94	2.73	1.64	0.31	1.64	1.74	0.21	0.31	1.14	0.30	0.25	3.87	1.07	1.42	2.20	0.21	0.15	0.11	1.028	0.098	0.056	0.188
Lachnoclostridium	0.75	0.03	0.17	1.02	7.34	5.61	6.23	6.92	5.71	5.07	2.96	5.20	3.48	3.39	5.02	4.66	1.60	3.67	2.60	0.34	0.57	0.73	1.716	0.0001	0.991	0.993
Phascolarctobacterium	0	0.16	0.08	0.14	0.55	0.03	0.20	1.40	1.90	0.20	3.67	2.38	1.52	3.94	3.34	1.42	4.00	5.69	1.95	3.63	3.62	2.97	0.619	<0.001	0.0004	0.007
Campylobacter	0	0.59	0	1.92	0.18	0.10	1.49	0.92	0.37	2.46	0.19	0.46	2.19	1.25	0.62	2.10	0.17	1.63	4.31	0.99	1.39	4.95	1.163	0.466	0.0007	0.956
Megasphaera	0.16	0	0.32	0	0.39	0.12	0	0.17	0.77	0.06	1.47	1.16	0.58	4.56	3.07	0.58	2.21	4.82	0.73	0.41	5.79	2.61	1.309	0.012	0.141	0.452
Christensenellaceae_R_7_group	0	0.38	0	0.27	0	0	0.40	1.06	1.24	0.65	8.27	7.09	5.72	1.64	4.45	2.55	1.66	1.33	3.54	5.09	3.41	3.39	1.370	<0.001	0.995	0.746
Prevotellaceae_NK3B31_group	0	0.30	0	0.22	0.36	0	0.45	1.26	0.09	0.22	1.75	0.57	0.70	2.39	1.57	0.50	4.16	2.09	2.08	3.85	3.18	2.18	0.622	<0.001	0.057	0.324
UCG_002	0.32	0	0.26	0	2.05	0.80	0.05	4.07	3.77	0.48	0.89	5.72	1.86	1.51	2.50	1.60	2.80	4.12	1.59	3.00	3.47	2.58	0.837	0.001	0.0014	0.008
Roseburia	0	0.15	0	0.32	0.01	0	0.33	0	0.88	0.19	2.06	1.01	0.99	4.05	0.82	0.65	2.42	2.98	1.44	2.17	1.82	1.73	0.605	<0.001	0.309	0.050
NK4A214_group	0.13	0	0.11	0	0.46	0.11	0.05	1.38	2.61	0.15	2.04	2.43	1.44	0.93	1.43	0.77	2.67	1.95	1.55	2.64	2.22	2.53	0.465	<0.001	0.061	0.035
Moraxella	0.50	2.87	0.08	0.03	0.05	0.06	0.04	0.01	0.02	0.05	0	0	0.02	0	0	0.01	0.02	0	0	0	0	0	0.042	<0.001	<0.001	<0.001
Veillonella	1.36	1.46	0.71	1.09	1.23	1.75	0.61	0.40	0.67	0.05	0.12	0.28	0.09	0.53	0.23	0.06	0.24	0.08	0.02	0.05	0.08	0	0.220	<0.001	0.012	0.294
Enterococcus	0.61	0	1.61	0.48	1.03	1.49	3.62	0.66	1.82	5.90	0.27	0.67	2.20	0.22	0.15	2.14	0.27	0.21	0	0.45	0.23	0	0.843	<0.001	0.005	0.002

Day of Life (DoL)	2				6			13			20			27			30			34			P-value			
**Sample Type**	**F + S**	**F**	**M**	**S**	**F + S**	**F**	**S**	**F + S**	**F**	**S**	**F + S**	**F**	**S**	**F + S**	**F**	**S**	**F + S**	**F**	**S**	**F + S**	**F**	**S**	**Pooled SEM**	**DoL**	**Type**	**DoL × Type**
Family_XIII_AD3011_group	0.02	0.01	0.03	0.01	0.07	0.01	0.01	0.60	0.32	0.05	2.39	0.92	0.94	0.69	1.49	0.60	1.50	1.32	1.62	1.78	1.42	2.15	0.389	<0.001	0.778	0.122
Desulfovibrio	0	0.02	0.02	0.12	0.93	0.77	0.38	1.36	0.70	0.46	0.88	0.97	0.74	1.29	1.50	0.62	1.00	0.79	0.79	0.86	0.61	0.64	0.229	0.006	0.052	0.082
Lachnospiraceae_NK4A136_group	0	0.16	0	0.09	0	0	0.14	0	0	0.10	0.76	0.28	0.34	0.36	0.80	0.32	3.96	3.43	1.88	1.56	1.68	1.55	0.534	<0.001	0.800	0.412
Colidextribacter	0.10	0.00	0.07	0.05	0.68	0.49	0.19	0.95	0.60	0.21	0.56	0.68	0.73	0.56	1.00	0.30	0.86	0.52	0.67	0.55	0.62	0.84	0.208	0.129	0.466	0.033
Ruminococcus	0	0.21	0	0.19	0.51	0.28	0.76	0.56	0.43	0.88	0.39	0.83	1.31	0.16	1.24	1.73	1.37	0.69	1.86	1.05	0.91	1.13	0.374	0.026	0.030	0.686
Rothia	0.14	0.26	0.07	0.06	0.08	0.15	0.04	0.03	0.09	0.02	0.02	0.05	0.04	0.02	0.01	0.01	0	0.01	0.01	0	0	0.01	0.024	<0.001	0.002	0.008
Subdoligranulum	0.15	0	0.12	0	0.22	0.17	0.02	0.67	0.37	0.19	2.08	0.54	0.33	0.62	1.38	0.78	1.16	0.85	1.19	1.92	1.27	1.53	0.435	0.0005	0.498	0.598
Mitsuokella	0.05	0.22	0.24	0	0.06	0.11	0	0.04	0.01	0.01	0.57	0.16	0.09	1.09	0.83	0.24	0.80	1.24	0.25	0.60	2.86	2.87	0.911	0.104	0.917	0.993
Oscillibacter	0.03	0	0.09	0	0.09	0.02	0	0.42	0.17	0	1.37	1.01	0.68	1.39	1.54	0.26	0.95	0.72	0.83	1.10	0.70	0.32	0.317	<0.001	0.073	0.260
Butyricimonas	0.01	0	0.02	0.06	0.67	1.03	0.18	2.95	1.25	0.65	0.56	0.93	0.33	0.50	0.82	0.47	0.09	0.15	0.37	0.01	0.05	0.04	0.334	<0.001	0.186	0.002
Coprococcus	0.02	0	0.04	0.06	0.07	0.22	0.04	0.19	0.12	0.24	1.94	0.40	0.31	1.56	0.81	0.54	1.82	0.52	1.47	1.20	0.87	4.00	0.463	<0.001	0.147	<0.001
Parabacteroides	0.23	0	0.43	0.31	1.32	0.76	0.44	0.72	1.16	0.48	2.98	0.58	0.10	0.17	0.54	0.09	0.71	0.48	0.64	0.66	0.65	0.49	0.408	0.074	0.105	0.029
Helicobacter	0	0.11	0	0.07	0.08	0	0.17	0.16	0.06	0.30	0.10	0.27	3.83	0.35	0.18	1.02	0.17	0.01	2.30	0	0	1.09	0.826	0.358	0.028	0.194
Agathobacter	0	0.13	0	0.08	0	0	0.14	0	0.04	0.08	0	0	0.08	0	0.09	0.22	2.02	1.10	0.50	1.92	1.71	1.88	0.362	<0.001	0.986	0.502
Terrisporobacter	0.19	0.06	0.14	0.55	0.15	0.04	0.09	0.11	0.04	0.54	0.23	0.47	0.47	0.10	0.17	0.30	0.61	0.35	1.05	0.90	0.39	0.58	0.274	0.066	0.186	0.846
Prevotellaceae_UCG_001	0.05	0	0.04	0.0	0.05	0.02	0	0.18	1.00	0.01	0.16	1.11	0.01	1.38	0.87	0.06	0.57	0.63	0.50	0.54	0.40	0.56	0.499	0.551	0.458	0.784
Tuzzerella	0	0.07	0	0.17	0.20	0.18	0.18	0.15	0.33	0.09	0.89	1.24	0.23	0.32	0.69	0.32	0.71	0.68	0.27	0.17	0.79	1.08	0.411	0.290	0.762	0.812
Romboutsia	0.10	0	1.01	0	0.16	0.05	0.04	0.23	0.31	0.37	0.28	0.32	0.27	0.11	0.42	0.20	0.15	0.17	0.41	0.10	0.05	0	0.170	0.238	0.0003	0.898
Blautia	0.05	0	0.04	0	0.11	0.16	0.04	0.07	0.10	0	0.49	0.20	0.26	0.14	0.21	0.87	0.52	0.39	1.33	2.36	0.61	4.00	0.333	<0.001	0.002	<0.001
Dorea	0.02	0	0.02	0.06	0.12	0.06	0.06	0.42	0.47	0.10	0.45	0.93	0.88	0.30	0.66	0.41	0.32	0.56	0.74	0.80	0.63	1.03	0.243	0.0007	0.854	0.599
Porphyromonas	0.04	0	0.01	0.07	0.14	0.01	0.57	0.05	0	0.68	0.01	0.01	0.01	0.01	0.01	0.23	0.01	0.01	0.35	0.03	0.01	0.06	0.246	0.788	0.152	0.841
Alistipes	0	0.04	0	0.03	0.58	0.25	0.15	1.76	1.18	0.31	0.30	0.69	0.13	0.62	1.29	0.18	0.24	0.42	0.28	0	0.03	0.05	0.279	<0.001	0.048	0.016
Lachnospiraceae_AC2044_group	0	0.01	0	0.01	0	0	0.01	0.05	0.01	0.08	0.31	0.38	0.76	0.08	0.03	0.10	1.86	1.60	0.64	0.90	0.92	0.70	0.313	<0.001	0.910	0.262

*Values are least squares means ± pooled standard error of the mean (SEM). Piglets were weaned on day 28 of life. Sample type: F, feces; M, meconium; S, swab; F + S, combined feces + swab sample. Proportional abundances are presented that represent >0.2% of all reads.
